# The potential use of mesenchymal stem cells-derived exosomes as microRNAs delivery systems in different diseases

**DOI:** 10.1186/s12964-022-01017-9

**Published:** 2023-01-23

**Authors:** Elham Oveili, Somayeh Vafaei, Haniyeh Bazavar, Yeganeh Eslami, Ehsan Mamaghanizadeh, Saman Yasamineh, Omid Gholizadeh

**Affiliations:** 1 Department of Pharmaceutical Science, Azad Islamic University of Medical Sciences, Tehran, Iran; 2 Department of Molecular Medicine, Faculty of Advanced Technologies in Medicine, Iran University of Medical Sciences, Tehran, Iran; 3 Infectious and Tropical Diseases Research Center, Tabriz University of Medical Sciences, Tabriz, Iran; 4 Faculty of Medicine, Mazandaran University of Medical Sciences, Sari, Iran; 5 Department of Biotechnology, School of Medicine, Tabriz University of Medical Sciences, Tabriz, Iran; 6 Department of Bacteriology and Virology, School of Medicine, Tabriz University of Medical Sciences, Tabriz, Iran; 7 Research Center for Clinical Virology, Tehran University of Medical Sciences, Tehran, Iran

**Keywords:** Non-coding RNA, Mesenchymal stem cells, Exosomes, Drug delivery system

## Abstract

MicroRNAs (miRNAs) are a group of small non-coding RNAs that regulate gene expression by targeting mRNA. Moreover, it has been shown that miRNAs expression are changed in various diseases, such as cancers, autoimmune disease, infectious diseases, and neurodegenerative Diseases. The suppression of miRNA function can be easily attained by utilizing of anti-miRNAs. In contrast, an enhancement in miRNA function can be achieved through the utilization of modified miRNA mimetics. The discovery of appropriate miRNA carriers in the body has become an interesting subject for investigators. Exosomes (EXOs) therapeutic efficiency and safety for transferring different cellular biological components to the recipient cell have attracted significant attention for their capability as miRNA carriers. Mesenchymal stem cells (MSCs) are recognized to generate a wide range of EXOs (MSC-EXOs), showing that MSCs may be effective for EXO generation in a clinically appropriate measure as compared to other cell origins. MSC-EXOs have been widely investigated because of their immune attributes, tumor-homing attributes, and flexible characteristics. In this article, we summarized the features of miRNAs and MSC-EXOs, including production, purification, and miRNA loading methods of MSC-EXOs, and the modification of MSC-EXOs for targeted miRNA delivery in various diseases.

**Graphical abstract:**

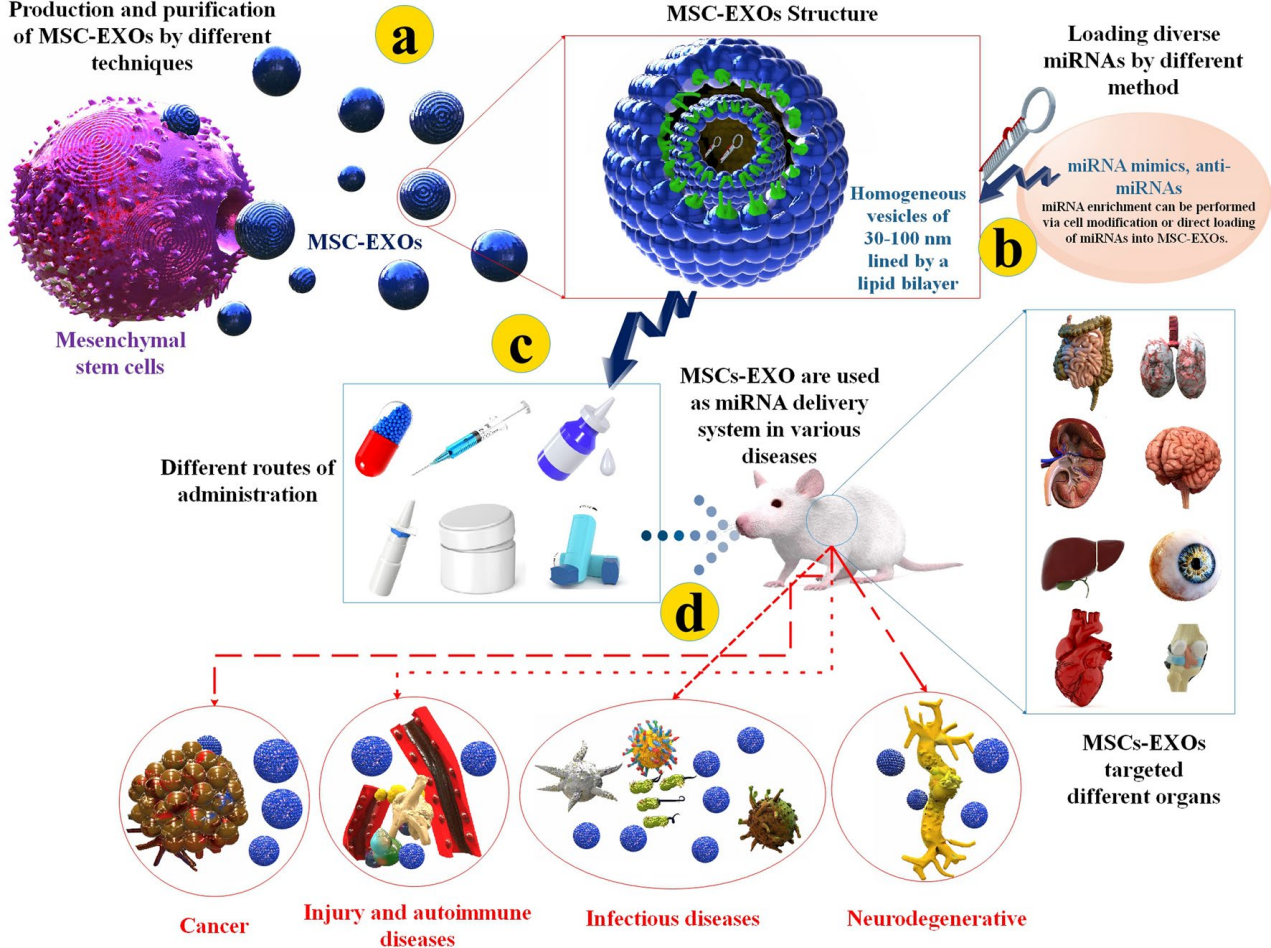

**Video abstract**

**Supplementary Information:**

The online version contains supplementary material available at 10.1186/s12964-022-01017-9.

## Introduction

MicroRNA (miRNAs) are short single-stranded non-coding RNA (ncRNA) that control and affect the expression of various genes [1]. About 30–60 percent of all mammalian proteins can be targeted via miRNAs, which are involved in different cellular and developmental procedures. MiRNAs control cell proliferation, differentiation, renewal, and apoptosis [2]. In addition, with the quick progress of next-generation sequencing (NGS), investigators have understood that miRNA interposition can alter associated physiological roles, causing inflammation of cell penetration, cancer, infectious diseases, neurological and immunological disorders, and other diseases [3–5]. Currently, some miRNA medications are undergoing clinical phases, and excellent advance has been made in the investigation and progress of miRNA medication patents and miRNA treatment. However, there are some obstacles to the clinical usage of miRNAs, including inconstancy in vivo and problems related to crossing biological barriers [6].

Exosomes (EXOs) are endosomal source membrane extracellular vehicles (EVs) with a dimension of about 30 to 150 nm secreted via types of cells. EXOs contained different compounds, including nucleic acid (such as miRNAs), proteins, enzymes, and lipids [7–9]. EXOs are natural biological materials with the intrinsic roles and protein machinery that safely and effectively deliver their load biomaterials among origin and target cells over long distances. EXOs play a crucial function in intercellular communication. EXOs are being progressively used as curative carriers. There is robust literature that EXOs therapy emerges as harmless with low immunogenicity [10–12]. Furthermore, EXOs are amenable to in vivo and in vitro loading of curative agents and membrane modifications to improve tissue-particular homing [13]. Several EXOs are obtained from various cells, such as mesenchymal stromal cells (MSCs) [14, 15]. MiRNA-loaded EXOs have been employed in multiple diseases. MiRNAs encapsulated in EXOs are usually attained via transfecting adipose tissue-obtained stem cells (SCs) and MSCs with the desired miRNA [16].

The properties of MSCs include the facility of production and isolation, low immunogenicity, harmless, lack of side effects, and efficient therapeutic method. Robust clinical assessments suggest that MSC-based therapies lack side effects, practicable and are efficient [17–19]. In addition, several investigations have obtained and identified EXOs from different MSC sources, such as bone marrow (BM), adipose tissues (AD), and umbilical cord (UC), approving their robust anti-inflammatory, anti-fibrotic and angiogenesis-regeneration capabilities [20]. MSC-EXOs have numerous exclusive features, including small dimensions, low immunogenicity, long-term circulating, sustained release, tumor-homing, tissue-particular homing, excellent permeation, and excellent biocompatibility. In novel investigations, researchers use MSC-EXOs as a vehicle to transport RNA, protein, and molecular medications to particular sections of the body to attain targeted therapy [21–23]. Up to now, about 150 miRNAs and more than 900 proteins have been recognized in loads of MSC-EXOs, leading to the modification of a diversity of actions in target cells through diverse pathways [24, 25]. However, difficulties, including carrier isolation and purification, maintenance and transport, medication loading method, and targeting, still exist [21–23].

In this review, we will summarize recent advances regarding the application and miRNAs loading techniques of MSC-EXOs, MSC-EXOs production method, and routes of administration of MSC-EXOs. Moreover, the transfer of different miRNAs through MSC-EXOs to treat diverse diseases will be highlighted.

## MiRNAs biogenesis and characteristics

**Fig. 1:**
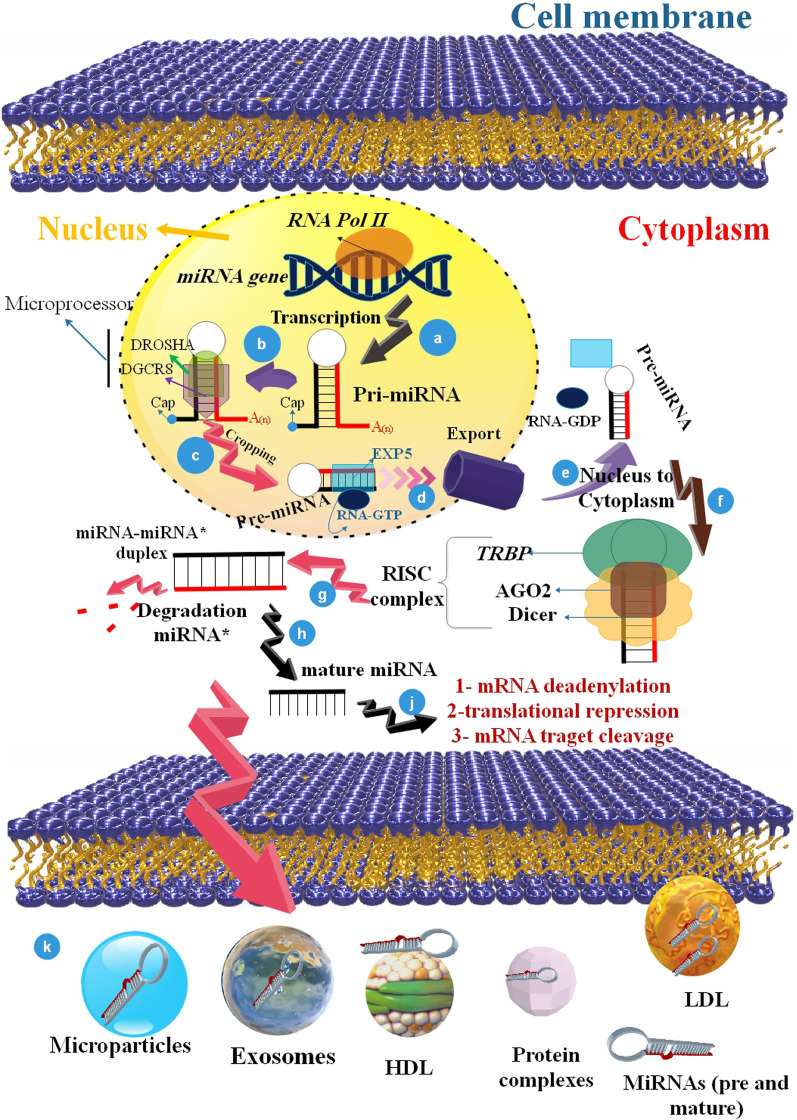
The biogenesis of miRNAs and their pathways to the target cell. **a-d** miRNA biogenesis starts in the nucleus. Pri-miRNA transcripts become pre-miRNA through the Drosha. Pre-miRNA is carried out of the nucleus through Exportin-5 into the cytoplasm. **e-j** MiRNA maturation is the elective loading of the functional strand of the small RNA duplex onto the RISC. miRISC induces the destruction of mRNA and translational inhibition via the interplay with the supplementary sequences in the 3′-UTR of target mRNA. **k)** MiRNAs are released in the extracellular environment or circulate through AGO2 protein, microvesicles, EXOs, high-density lipoprotein (HDL), and low-density lipoprotein (LDL) particles

MiRNAs are having the size of 18 ~ 25 nucleotides (nt) in length that post-transcriptionally up or down-regulate genes by connecting to the 3′-untranslated regions (3′-UTRs) of mRNAs [26, 27]. Briefly, the biogenesis of miRNAs begins with their transcription via RNA polymerase II (RNAP II), leading to an early transcript recognized as pri-miRNA [28, 29]. Afterward transcription, the ordinary hairpin-loop secondary construction existing in pri-miRNAs is detected and cleaved via the microprocessor complex (formed via DGCR8 and Drosha). The produced precursor miRNAs (pre-miRNAs) are transported to the cytoplasm and subsequently processed via the Dicer nuclease to produce a double-stranded RNA. The mature miRNA sequence is elected via Ago2 and involved in the RNA-induced silencing complex (RISC) to use its regulatory function [4, 30, 31]. Each miRNA can bind to several mRNAs. The role of miRNAs is to suppress the protein synthesis of protein-coding genes by suppressing the translation of targeted mRNA or through mRNA degradation. Furthermore, miRNAs can activate the translation of targeted mRNAs, switching between translation suppression and triggering in coordination with the cell cycle. In addition, miRNAs have been shown to play a crucial function in many biological activities, such as metabolism, immunity, cell development, apoptosis, differentiation, and signal transduction [32–34]. The deregulation of miRNAs in disease situations can be used as possible curatives via either miRNA substitution treatment utilizing miRNA mimics or suppression of miRNA role via antagomiRs [35, 36] (Fig. 1).

## MiRNA delivery system

**Fig. 2:**
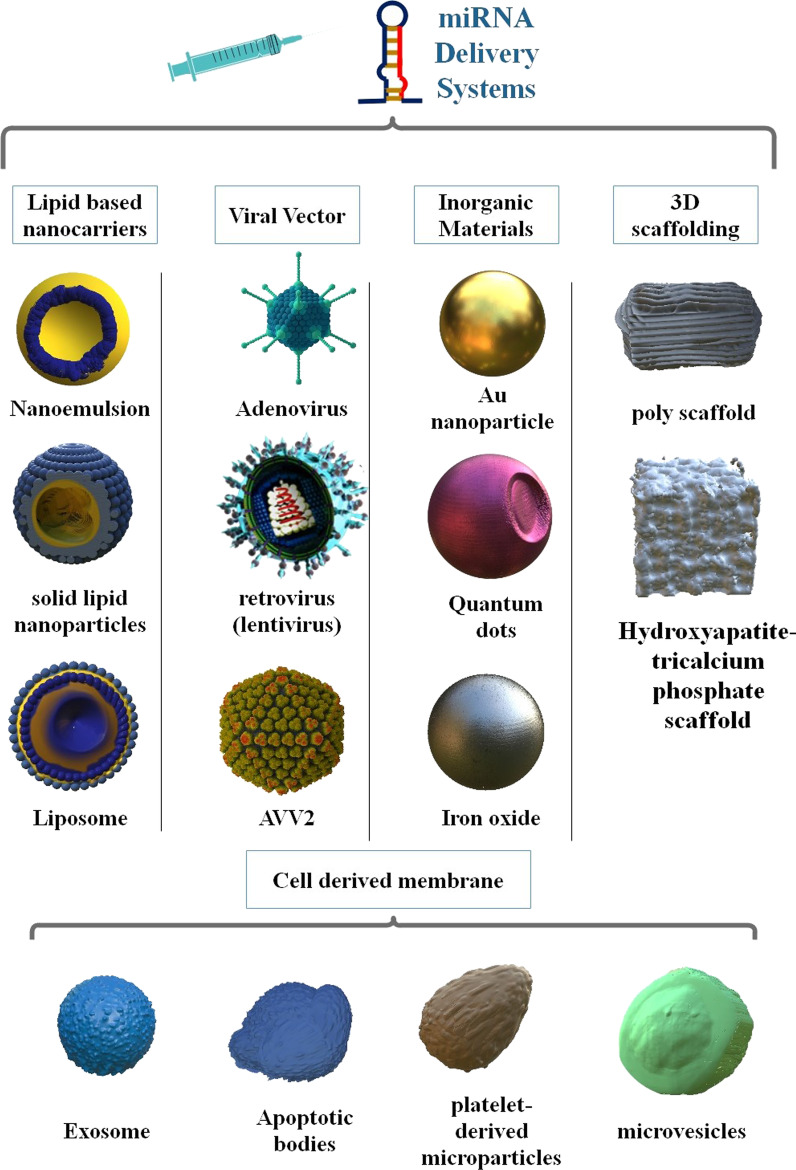
Various miRNAs delivery systems, including organic and inorganic nanoparticles, viral vectors, and extracellular vesicles. Inorganic NPs, including lipid-based nanoparticles, polymeric carriers/dendrimer-based, cell-isolated membrane vesicles, and 3D scaffold-based carriers

Notwithstanding the excellent capability of miRNA in treating various diseases, it has several limitations, which must be resolved. Firstly, because of the negative charge of miRNAs, they are hard to penetrate the cell. Moreover, miRNAs are unstable in vitro due to, destruction by nucleases, and immunotoxicity. Hence, the advance of harmless and effective miRNA delivery methods is highly important to the most effective use of miRNA as therapeutic agents [37]. Chemical changes have been a principal method for antagomiRs to inhibit their destruction via nucleases in cells and in vivo. Viral and non-viral carriers have been produced to transfer miRNA mimics or antagomiRs for the targeted treatment of various diseases. Viral carriers contain viruses that are altered to be replication-deficient, however, they can be utilized to carry nucleic acid for expression [38]. Moreover, different types of nanoparticles (NPs) propose unique chances for cell-particular controlled transfer of miRNAs to treat diseases. MiRNA-encapsulated in NPs has been offered, by NPs potential to protect the packed factor from the extracellular environment, thereby decreasing destruction, and increasing circulation time and selective accumulation [39, 40]. Notwithstanding the significant progress and achievements in their formulation, preparation, and efficiency of synthetic miRNA delivery systems, there is a developing acknowledgment that nature has particulates with some of the highly favorable properties of miRNA delivery systems, including immunologically inert, an inherent tropism that causes exceedingly selective and effective entrance into special host cells, stability in the different condition, and diverse therapeutic cargos and that such particulates should be used as miRNAs transfer carriers. Red blood cells (RBCs), bacteria, lymphocytes, extracellular vesicles (EVs), and EXOs are examples of natural miRNA delivery systems [41–44] (Fig. 2).

## MSCs-EXOs biogenesis and characteristics

MSCs are recognized as the most desirable origin for curative EXOs. This can be ascribed to the harmlessness of MSCs, the possibility of the generating MSC‐EXOs as an off‐the‐shelf production, and lack of tumorigenic possibility of MSC-EXOs. Therefore, the use of MSC‐EXOs substitute MSCs in a diversity of diseases, such as cancer, autoimmune diseases, tissue injury, neurodegenerative diseases, infectious diseases, ocular diseases, and dermal diseases [45, 46]. Moreover, MSCs are the only recognized cells able to generate EXOs at a large scale. Large scale generation can be attained via obtaining and, culturing MSCs in vitro [47].

They have been classified based on EXOs dimensions, compounds, and formation mechanism in the last years. There are recognized subtypes of EVs comprising microvesicles (MVs), apoptotic bodies (ABs), and EXOs. In addition to EXOs, other membrane vesicles generated via cells contain plasma membrane-budded MVs and ABs [48]. In further investigations, Wnt and mTOR pathways, which lead to overexpression of β-catenin, are presented as main controllers essential both for MSC-EXO discharge and as well as supporting the self-renewal of MSCs [49, 50].

**Fig. 3:**
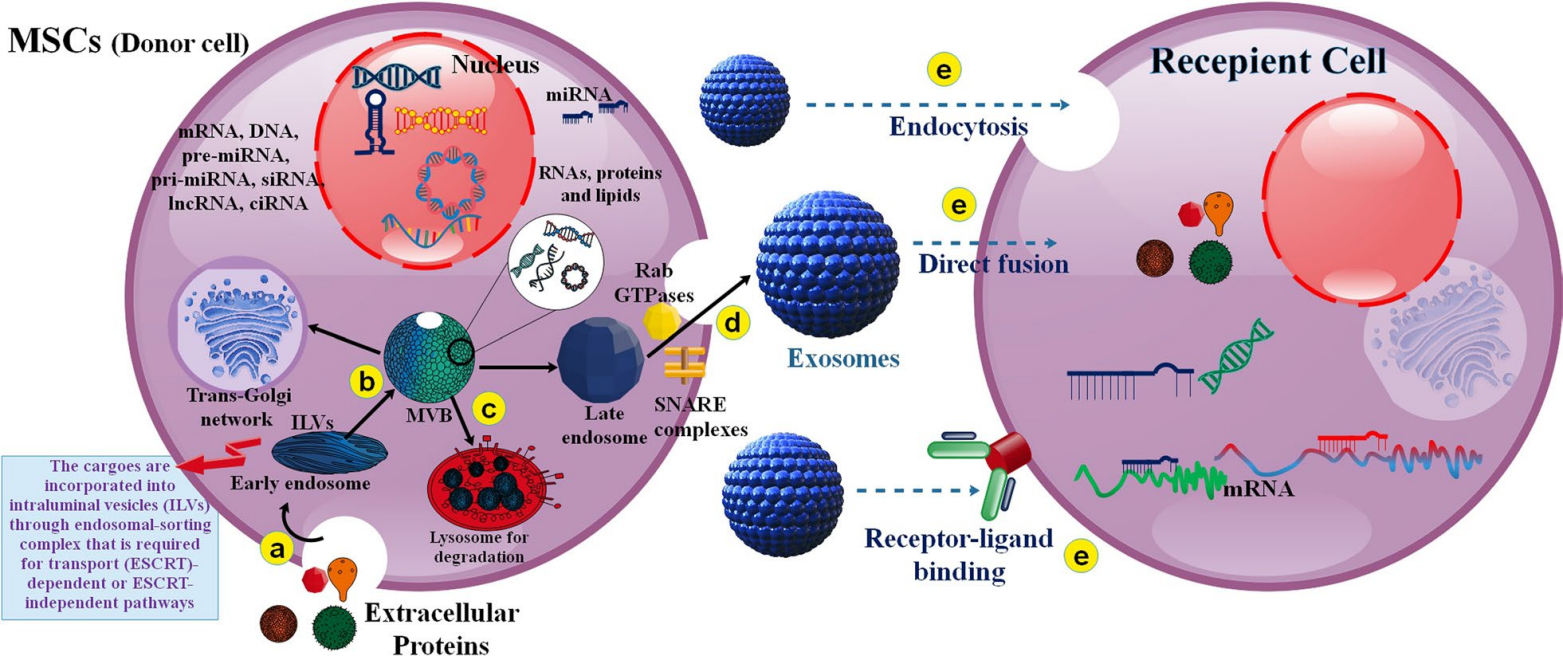
The biogenesis of MSC-EXOs and their pathways to the target cell. **a** EXOs biogenesis starts within the endosomal system. Within the endosomal system, internalized loads are encapsulated into early endosomes, **b** which then mature into late endosomes or MVB. Different compounds are also transported from the trans-Golgi network and presumably from the cytosol. **c** In addition, MVBs can be transported to lysosomes for decay or **d** move along microtubules to amalgamate with the cell membrane and discharge EXOs into the extracellular environment. MVB amalgamation with the membrane is a fine-tuned process, which needs various vital agents, including Rab GTPases and SNARE complexes. **e)** EXOs intercede their effects on the target cells via three essential methods: 1- recipient cell receptor interceded signal amplification, 2- direct connecting to the plasma membrane and fusion, and 3- endocytosis

**Fig. 4:**
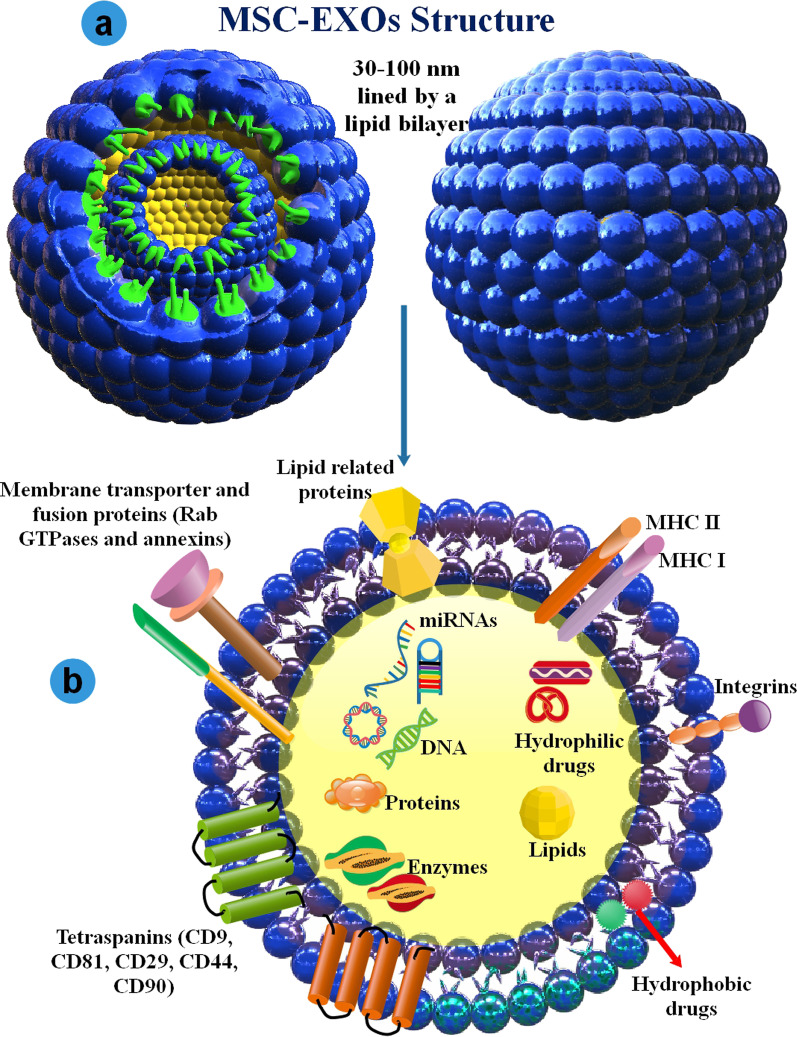
**a** 3D schematic structure and contents of MSCs-EXOs. **b** EXOs usually contain dynamic loads, including different proteins, enzymes, lipids, RNA (such as miRNAs), and DNA. Moreover, MSC-EXOs carry and deliver different hydrophobic and hydrophilic drugs

The mechanism of EXO generation during EXO biogenesis includes the beginning; the plasma membrane is internalized to form an endocytic vesicle called an early endosome, followed by early endosome to late endosome transformation. The budding of late endosomal membranes results in forming intraluminal vesicles (ILVs) within large MVBs [51]. Comprehensively, EXOs biogenesis is controlled via two distinct molecular pathways, comprising endosomal sorting complex required for transport (ESCRT) machinery- affiliate and ESCRT-autonomous [52, 53]. EXOs discharge relates to transfer and plasma membrane fusion of the secretory MVBs subsequent internal budding of ILVs, which needs multiple critical agents, such as molecular switches (small GTPase), cytoskeleton (microtubule and microfilament), molecular motors (dynein and kinesin) and the membrane fusion proteins (SNARE complex). Rab GTPase is the most crucial agent, with more than 70 subtypes placed on the surface of membranes, where they can control vesicle traffic, such as budding, motility, and fusion. In addition, Rab35 localizes to the surface of oligodendroglia cells in a GTP-related method, regulating the docking of endocytic vesicles with the membrane. MVBs and plasma membranes can amalgamate through intermediation via Rab and the corresponding effector on the MVB membrane [54]. EXOs are taken up via the recipient cells by direct connecting to the plasma membrane, binding to the host-receptor, and by endocytosis. EXOs comprise proteins, DNA, RNA (such as miRNA, lncRNA, mRNA, tRNA, and circRNA), and cholesterol (Fig. 3 and Fig. 4) [55, 56].

## MSC-EXOs preparation by different techniques

To ensure the biological function of EXOs, a standardized engineering procedure, including a process by good manufacturing practice (GMP), of EXOs is essential. As EXOs are released via cells, a generation method could be created by a large-scale cell cultivation technique. EXOs in clinical experiments need to comply with GMP. Three crucial challenges are prevalent in GMP for EXOs, including upstream of cell culture, downstream of the purification method, and EXOs quality control [57, 58].

The minimum prerequisites for utilizing EXOs as medication delivery systems are an excellent efficiency cell origin of EXOs and a reproducible, scalable purification procedure to form a highly determined population of EXOs. Though most cell kinds generate EXOs, the quantity of EXOs generated via each cell kind is highly changeable. EXOs generated via cultured cells are ordinarily obtained from the medium conditioned for 1–7 days via the cells utilizing either medium without serum or medium with the serum that had been discharged of MVs [13]. Investigators have applied scaffolds, spherical culture, a mercantile hollow-fiber bioreactor method, stirred suspension bioreactors, perfusion-based bioreactors, and microcarrier-based 3D culture techniques to attain more significant EXOs generation [59, 60].

### MSC-EXOs produced by 3D cultures

MSCs cultivated in two-dimensional (2D) traditional tissue culture polystyrene flasks can lead to low efficiency, restricting their clinical use. 3D culture might be an effective method for the enhanced biological purposes [61, 62]. The advantages of using the three-dimensional cultivation method include the culture, a multitude of cells the high efficiency of EXOs. Compared with 2D culture, the EXO generation of 3D culture is 19.4 times higher. In addition, 3D-EXO cultures are more concentrated in the harvested supernatants (15.5-fold) than 2D-EXOs, which results in a greater EXOs collection yield [63]. Scaffold-free and scaffold-based culture methods produced of natural or synthetic substances are the two broad groups of 3D culture methods. There are various scaffold-based 3D culture techniques, including hydrogels and solid scaffolds. For example, researchers showed that contrasted with 2D culture, human BM-MSCs cultured in the 3D collagen scaffolds produced further EXOs with enhanced repair role in rats afterward intracranial injury. In another investigation, researchers showed that scalable microcarrier-in 3D cultures could twofold the accumulation of MSCs and efficiency more EXOs than 2D cultures [62, 64, 65].

### MSC-EXOs produced by bioreactor production methods

**Fig. 5:**
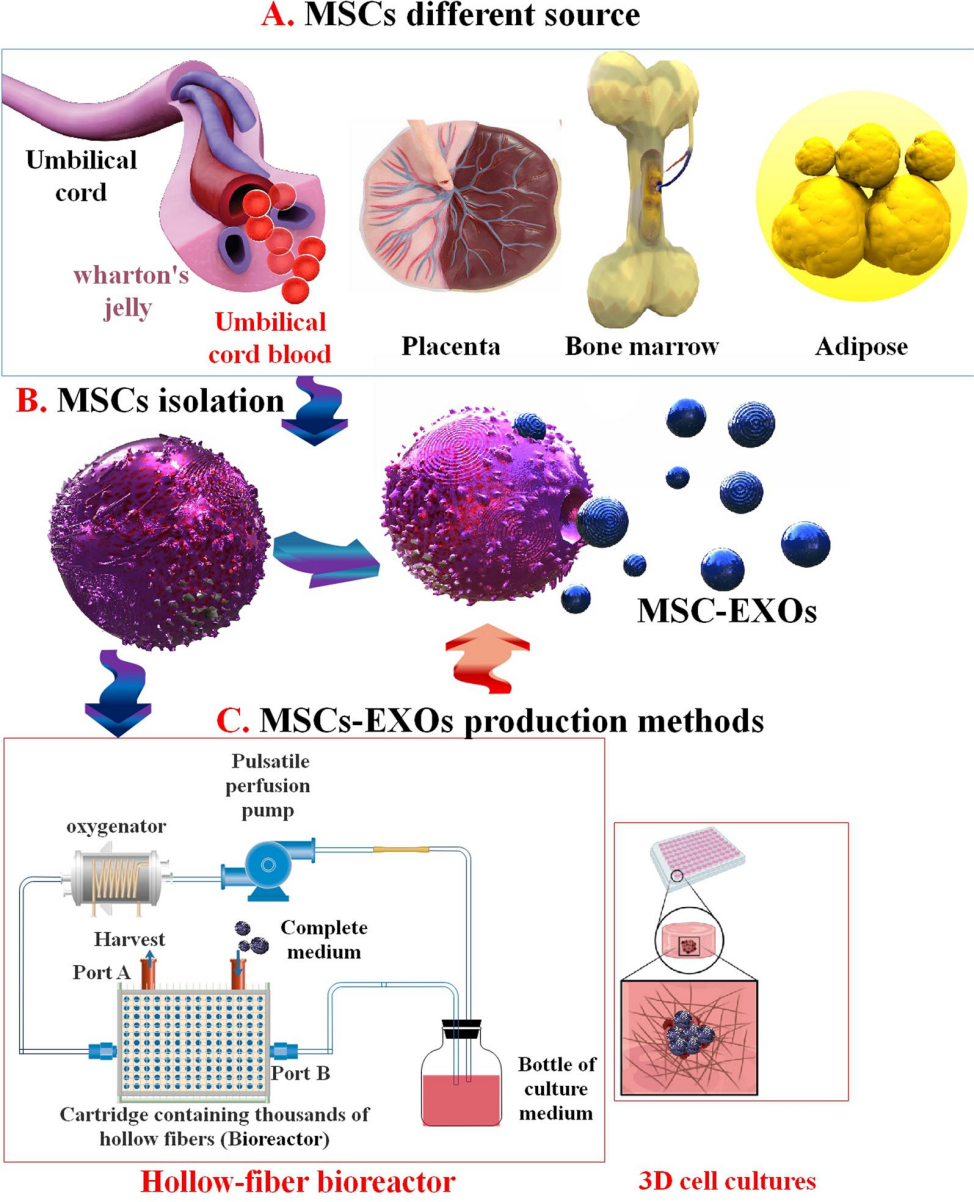
MSCs have been isolated from different sources (**A** and **B**). Exosomes are produced by various methods from diverse types of MSCs (**C**). The standard diagram of the HFB-based 3D culture method. The technique was prepared of a pulsatile perfusion pump, an oxygenator, a cartridge comprising thousands of hollow fibers, a bottle of culture media, and the linking tube

The conventional 2D culture technique to generate EXOs from adherent cells does not allow for a sustained generation of large amounts of biological production and thus prevents their usage for clinical trials. On the other hand, bioreactor methods remove this limitation while preparing the needful milieu to preserve excellent cell viability and homeostasis. EXO manufacture according to a bioreactor method offers numerous advantages where scalability, decreased manual manipulation, and simple monitoring and regulating of culture parameters can be attained. Moreover, the usage of a bioreactor can enhance the translational competency of bio therapeutics as this milieu is an improved presentation of the cell–cell interplay found in vivo as compared to the flask-based technique [64, 66]. The commercial hollow-fiber bioreactor (HFB) method has been used efficiently for the mass production of EXOs. The HFB from Fiber Cell Systems allows for seeding large quantities of adherent cells based on its HFB technology which subsequently enhances the cell seeding surface area (an average-sized cartridge provides 4000 cm^2^ of surface area) [64, 67, 68]. Novel investigations have used more straightforward methods (such as flat-plate bioreactors), permitting a more accurate approach for comprehend and restraining flow-derived shear stress. However, still absence the adjustability essential for dynamic construction and experimental investigation requirements [69]. Moreover, stirred suspension bioreactors (SSBs) have been efficiently utilized to scale up MSCs manufacture. These techniques are partly simple to perform, propose scalability benefits, supply an excellent capacity for cell development, can help adherent cell development with the addition of microcarriers or accumulations, and allow a homogenous culture microenvironment because of continuous stirring [70] (Fig. 5).

## Separation and purification of MSC-EXOs

MSC-EXOs as a medication delivery system in vivo, a harmless, effective, and trustworthy purification technique is essential. Presently, there is numerous generally utilized techniques for EXO filtration [13]. Various recognized methods, including differential ultracentrifugation, density gradients, sedimentation, ultra-filtration, and size exclusion chromatography, have been used for EXO isolation [71]. The most popular utilized separation technique for EXOs is differential centrifugation, where an enhancing centrifugal force from 200 × g to 100,000 × g is used to primarily deplete the media of bigger units and cell remnants before finally precipitating EXOs at 100,000 × g [13, 72]. Newly, some commercial kits have been offered to separate EXOs for several targets [73]. These kits are more significant to ultracentrifugation because of being less time-consuming, less method sensitive, and higher compatibility with confined content of samples [71, 74, 75].

Newly, microfluidic methods have been used in EXOs isolation. The attributes of the microfluidic-based EXOs isolation methods, such as high surface-area-to-volume proportion, reduce use of samples, quick analysis, laminar flow, and simple implementation method, make them appropriate for EXOs isolation with an excellent recovery level and purity for clinical uses [76]. To efficiently exploit the dimensional difference among EXOs, other types of EVs, and cellular debris, researchers manufactured a poriferous silicon nanowire-on-micropillar “nano-trap” produced from ciliated micropillars. This manufactured microfluidic machine more favorably traps EXOs with a size of 40–100 nm, while purifying proteins, larger EVs, and cellular debris. Furthermore, trapped EXOs can be recovered by solving the porous silicon nanowires in phosphate-buffered saline buffer [77].

**Fig. 6:**
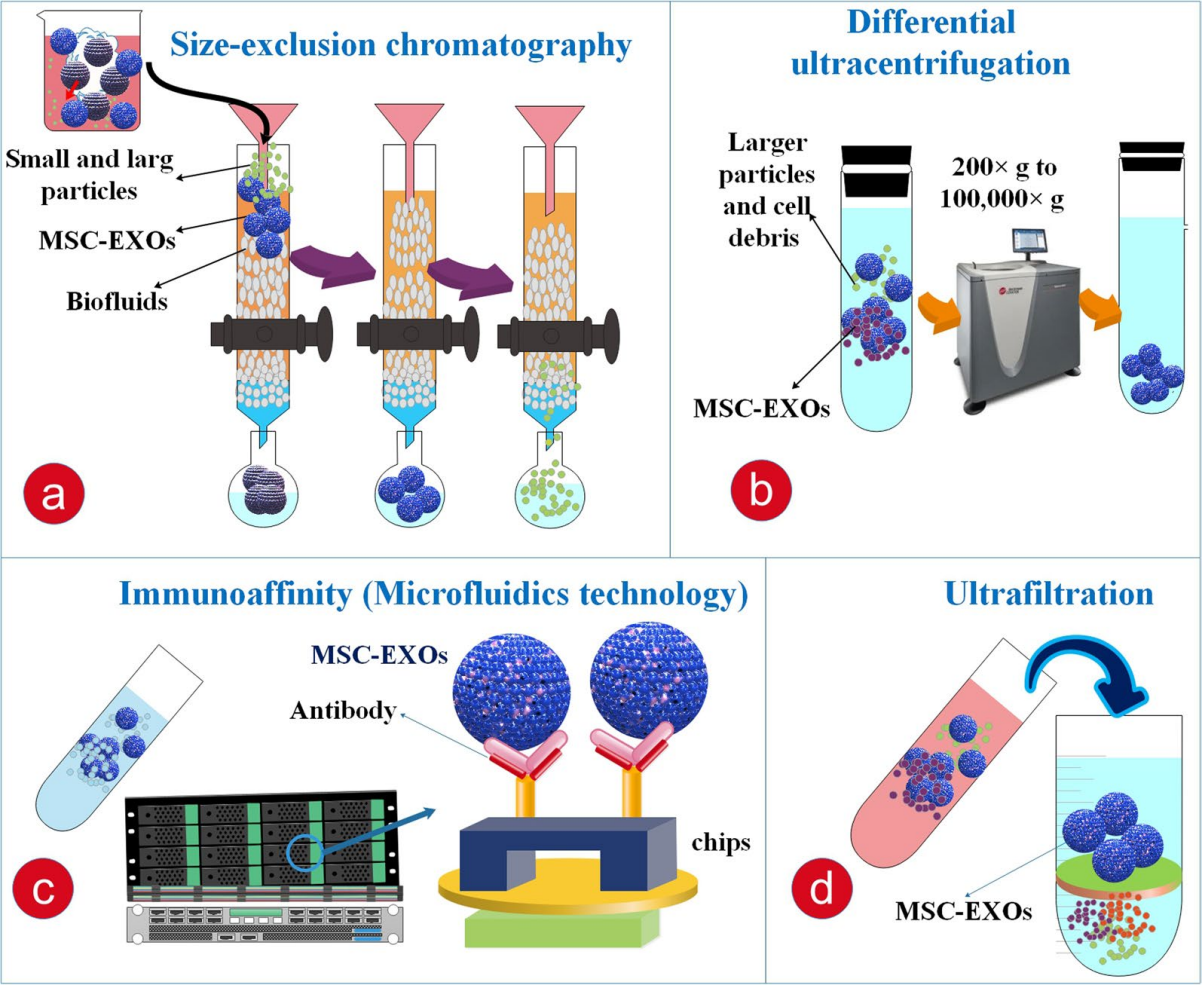
Traditional and new techniques of EXO purification. EXOs are classified into three main kinds associated with their location of source, density, expression of markers, and dimensions. Traditional techniques of EXO separation contain differential ultracentrifugation and size-exclusion chromatography. **a** Size-exclusion chromatography utilizes biofluids as a mobile state versus a porous static state to differentially elute molecules with a reverse speed relative to their dimension. **b** Differential ultracentrifugation depends on the isolation of EXO subpopulations through progressively higher speed rates. Polyethylene glycol (PEG)-based sedimentation uses a solution to ease a polymer-encapsulated vesicle collection in large amounts. **c** Immunoaffinity absorption utilizes antibodies targeted versus exosomal surface proteins to separate particular vesicle populations. The microfluidics (MF) method utilizes chips with particular antibody-interceded connections to capture EXOs effectively. **d** Ultrafiltration is associated with a filter of a particular pore size that forms a vesicle-rich filtrate particular to the favorable dimensions [164, 165]

Among the several separation techniques, tangential flow filtration (TFF) has been offered as the good technique for the large-scale production of EXOs. The TFF methods accessible for GMP are now in application and offer confirmed procedures and GMP documents. This method was primarily presented in 2010 for the separation of EXOs based on size and was progressively used for EXOs purification or concentration in different investigational settings. More significantly, novel investigation displayed the superior efficiency and activity of EXOs separated via TFF compared with those separated through ultracentrifugation. The great-purity separation of EXOs is attainable with extra diafiltration by TFF with suitable pore dimensions and parameters, such as transmembrane pressure, flow rate, and diafiltration factor [78] (Fig. 6).

## MiRNAs loading methods in MSC-EXOs

EXOs could be packed with different therapeutic agents either in vivo during their biogenesis or in vitro in isolated EXOs. However, the progress and improvement of effective use of EXO loading methods are presently restricted via our little knowledge of EXO biology, construction, and biogenesis and an absence of EXO-associated investigation and progress tools [79, 80]. To load miRNAs in EXOs, two techniques have been used. A cell culture overexpressing the miRNA of interest is produced, leading to enhanced miRNA expression and EXO discharge with the entrapped miRNA. Another method is separating EXOs and loading them with miRNAs [16]. Other methods of extracellular or in vitro MSC-EXOs drug loading includes: medication co-incubation, electroporation, acoustic processing, lipofection, sonication, squeeze technique, extrusion, saponin-helped loading, and freeze–thaw cycles. However, these techniques also have drawbacks, including the accumulation of EXOs, exosomal membrane damage, toxicity to target cells, and extreme separation and purification stages [81–85].

Another method for MSC-EXOs loading is to combine medications into EXOs during their biogenesis. This method is mainly related to the payload that cannot be encapsulated onto isolated EXOs, including cytosolic and transmembrane proteins or extraordinary molecular weight RNA, including mRNA [13, 86, 87]. A relationship matrix analysis investigation demonstrated a weak correlation between the RNA amount of MSC-EXOs and the principal MSCs, showing that miRNAs were electively packed into the EXOs. It has been proposed that RNA-binding proteins (RBPs), including hnRNPA2B1 and hnRNPA1 per se, connect to miRNAs and control their elective loading into EXOs. The endosomal sorting complex required for transport (ESCRT) has a significant function in loading protein-miRNA compounds into EXOs. Moreover, ESCRT-autonomous pathways, including ceramide–interceded mechanisms, have been demonstrated to be implicated in load sorting into EXOs. Additional study is required to discover the particulars of the elective miRNA loading method [88]. Moreover, RBPs and cell priming (such as hypoxia and inflammatory cytokines) methods can also be used to regulate the miRNA load of EXOs. Methods including sonication, electroporation, and CaCl2-heat shock can pack the miRNAs per se into the EXOs [89].

## MSCs-EXOs routes of administration

**Fig. 7:**
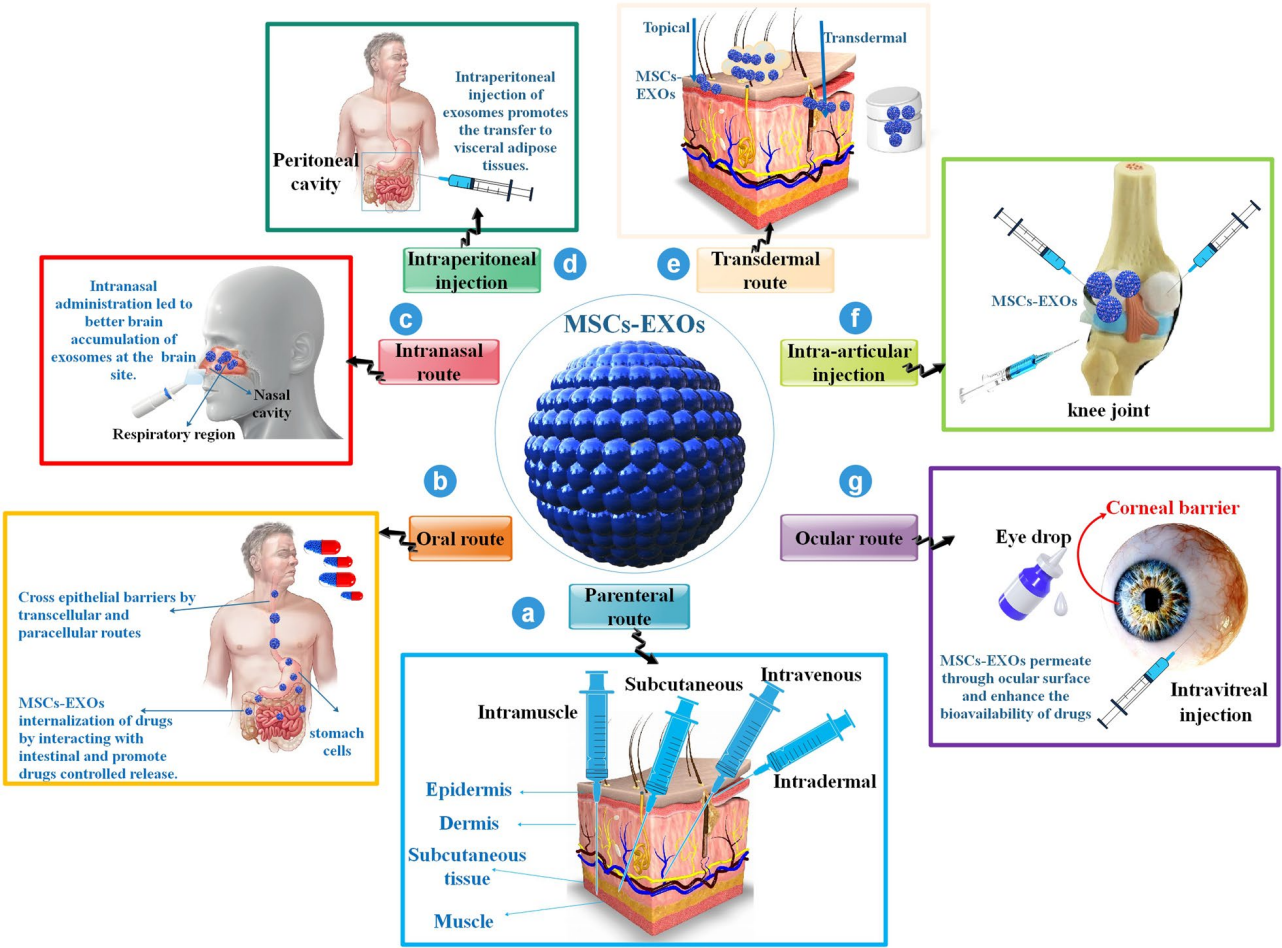
Diverse ways of administration utilized in MSC-EXOs delivery, including **a** the parenteral administration comprises; the three most common ways are intramuscular (i.m.), intravenous (i.v.), and subcutaneous (S.C.) administration; **b** Oral administration is a more straightforward and non-invasive method, although the transported EXOs must remain stable pass via the gastrointestinal tract [104]; **c** Intranasal injection resulted in improved brain aggregation of EXOs at the damaged brain location, compared to i.v. administration; **d** Intraperitoneal injection of EXOs is another injection method, with widespread EXO distribution capability [97]; **e** Regarding dermal injection routes, administration into the dermis, recognized as intradermal administration, and topical use are other easy choices to remedy dermal diseases; **f** Intra-articular administration next to the injured tissue is utilized for rheumatoid arthritis, leading to more effectiveness tissue repair because of the direct use of the drugs to the injured region; **g** MSCs-EXOs permeate through ocular surface and enhance the bioavailability of therapeutic agents

Choosing the best appropriate MSC-EXOs injection method, containing the dose injected, and considering the constancy, biological distribution, and toxicity of delivered EXOs is one of the main to attain effective cell-free treatment [90]. Diverse methods of administration utilized in MSC-EXOs have been studied, and it was defined that the route of administration had a significant effect on the preparation design (Fig. 7).

### Parenteral route

The parenteral administration comprises; the three most common ways intramuscular (IM.), intravenous (IV), and subcutaneous (S.C.) administration. It allows the direct administration of the drugs in systemic blood circulation. It is usually utilized when the drug is removed from the system by initial crossing metabolism or drug destruction in the stomach and gastrointestinal tract [91–93]. However, this medication route of injection has unique problems, including the aggregation of EXOs in non-targeted organs, usually the liver, spleen, and lungs, and rapid elimination from the organism [94]. For example, in bleeding into the brain tissue of a rat, DiI-labeled MSC-EXOs attained brain, liver, lung, and spleen afterward, IV administration. Biological distribution of systemically injected EXOs is an active procedure: a fast stage of delivery in the liver, spleen, and lungs within about 30 min upon injection is followed by a removal stage by hepatic and renal processing, eliminating EXOs in 1 to 6 h afterward injection [95]. In another study, Yang Zhou et al. showed that the topical drug of smearing human adipose-MSC-EXOs (hAD-MSC-EXOs) is more useful in improving skin injury regeneration than the S.C. administration of hAD-MSC-EXOs. For systemic injection, local injection composed treatment with the IV administration of hAD-MSC-EXOs presented an extra advantage over either therapy alone to improve cutaneous repair [96].

### Intraperitoneal delivery

Intraperitoneal injection of EXOs is another injection method with widespread EXO distribution capability. Moreover, researchers showed that intraperitoneal administration of AD-MSC-EXOs leads to an immunomodulatory influence on autoimmune type 1 diabetes, enhancing regulatory T-cell population and their production unchanged in lymphocyte proliferation [97].

### Intra-articular injection

Intra-articular administration next to the injured tissue is utilized for rheumatoid arthritis, leading to more effective tissue repair because of the direct use of the drugs to the injured region. For example, researchers showed that 12 intra-articular administrations per week of hMSC-EXOs were efficient in regenerating osteochondral lesions of the talus in rats [98]. Another investigation showed that the amalgamation of MSC-EXOs and hyaluronic acid injected at a clinically admissible frequency of 3 intra-articular administrations can improve stable and functional cartilage restoration in a rabbit after traumatic cartilage defect model when compared with HA alone [99].

### Intranasal route

Intranasal injection resulted in improved the brain aggregation of EXOs at the damaged brain location, compared to IV administration. This injection route is an appropriate choice when the purpose is to pass the blood–brain barrier, preventing EXO disintegration in other organs and supporting aggregation in the brain, leading to improved neuroprotective efficacy [100, 101].

### Transdermal route

Regarding dermal injection routes, administration into the dermis, recognized as intradermal administration, and topical use are other easy choices to remedy dermal diseases. The topical use of EXOs released via MSC-EXOs on the skin is a novel and interesting subject in the pharmaceutical. Newly, researchers showed that the topical use of human umbilical cord blood MSC-EXOs (hUC-MSC-EXOs) on ex vivo human skin, led to an enhanced expression of skin extracellular matrix (ECM) genes and therefore helped to rejuvenate the skin. In addition, the penetration and effectiveness of EXOs can be additionally enhanced by augmenting skin penetrance [102, 103].

### Oral route

Oral administration is a more straightforward and non-invasive method, although the transported EXOs must remain stable and pass via the gastrointestinal tract [104]. The oral route, similar to other routes, has been used to inject chemotherapeutic medications, including curcumin, leading to 3–fivefold greater amounts of curcumin in different organs compared to without encapsulated drug delivery [105]. Newly, an immune tolerance mechanism interceded via free light chain-covered, antigen-particular, miR-150-EXOs that affect the antigen-offering cells proved more efficient afterward oral injection [106].

### Ocular route

Ocular injection methods, comprising subconjunctival, intravitreal, and intraocular injection, despite being invasive and disturbing injection routes, have been used to treat diabetic retinopathy (damaging the retina) complications with progressive consequences. MSC-EXOs were as effective as transplanted MSCs in restricting the size of eye damage and inflammation. Immediately afterward, intravitreal administration of MSC-EXOs, because of nano-size, distributed quickly all over the retina and considerably decreased retinal injury and inflammation. MSC-EXOs effectively delivered trophic and immunomodulatory agents to the inner retina and effectively improved survival and neuritogenesis of damaged retinal ganglion cells [107–109].

## Different types of MSC-EXOs as miRNAs delivery systems in different diseases

MSC has been effectively isolated from various origins such as bone marrow (BM), adipose tissue (AD), Wharton’s Jelly (WJ), umbilical cord (UC), placenta, the dental pulp (DP), or amniotic fluid (AF) among others [110]. BM-MSCs have the benefits of less infection amount of pathogenic microorganisms, constant biological efficiency, low immunogenicity, and a large number of feasible passages [74]. BM-MSC-EXOs could speed up the production and migration of endothelial cells and osteoblast cells, improving angiogenesis, and osteogenesis to improve fracture repair [111]. In another study, researchers showed that hBM-MSC-EXOs therapy considerably decreased liver fibrosis in rats [112]. UC-MSCs can be obtained via a non-invasive strategy and easy cultured, thus offering their advantage over other types of MSCs for therapeutic objectives. Because of their unique characteristics, such as self-renewal, multipotency, and availability simultaneous with their immunosuppressive capability and lesser ethical worries, UC-MSCs treatment is defined as hopeful curative choices in cell-based treatments [113]. HUC-MSC-EXOs, acquired by extensively expanding hUC-MSCs in vitro, is convenient to extract, store, and transport, lower in immunogenicity, and better in biocompatibility. Over the past decades, it has been found that hUC-MSC-EXOs are mainly involved in improving tissue regeneration via delivering proteins, lipids, RNAs, and DNAs, which increase the progress of “cell-free therapy” [114]. The human body is abundant in AD tissue and, therefore, contrasted with UC-MSCs and BM-MSCs. AD-MSCs are plentiful with widespread origins and have extraordinary separation efficiency. However, AD-MSCs have challenging needs for storing situations and have low immunogenicity, which has limited their clinical use. The membrane construction of EXOs is relatively constant, and AD-MSC-EXOs can be stored for long times; hence, AD-MSC-EXOs has produced enhanced attention from investigators [74, 115]. AD-MSC-EXOs used preservative influences versus radiation-stimulated brain damage via reducing oxidative stress injury and decreasing inflammation and microglial penetration [116].WJ-MSCs, a mucosal-linked tissue of the UC, may be improved alternatives to BM-MSCs or AD-MSCs since WJ-MSCs are younger and preserved from injuries caused by senescent, environmental toxins and diseases [117].

MSC-EXOs can be an effective transfer mechanism for miRNAs. It's been investigated widely as a manner to release miRNAs in a controlled and targeted method to treat different cancers, neurodegeneration, autoimmune disorders, infectious diseases, and other diseases.

### MSC-EXOs as miRNA delivery system in cancer

**Fig. 8:**
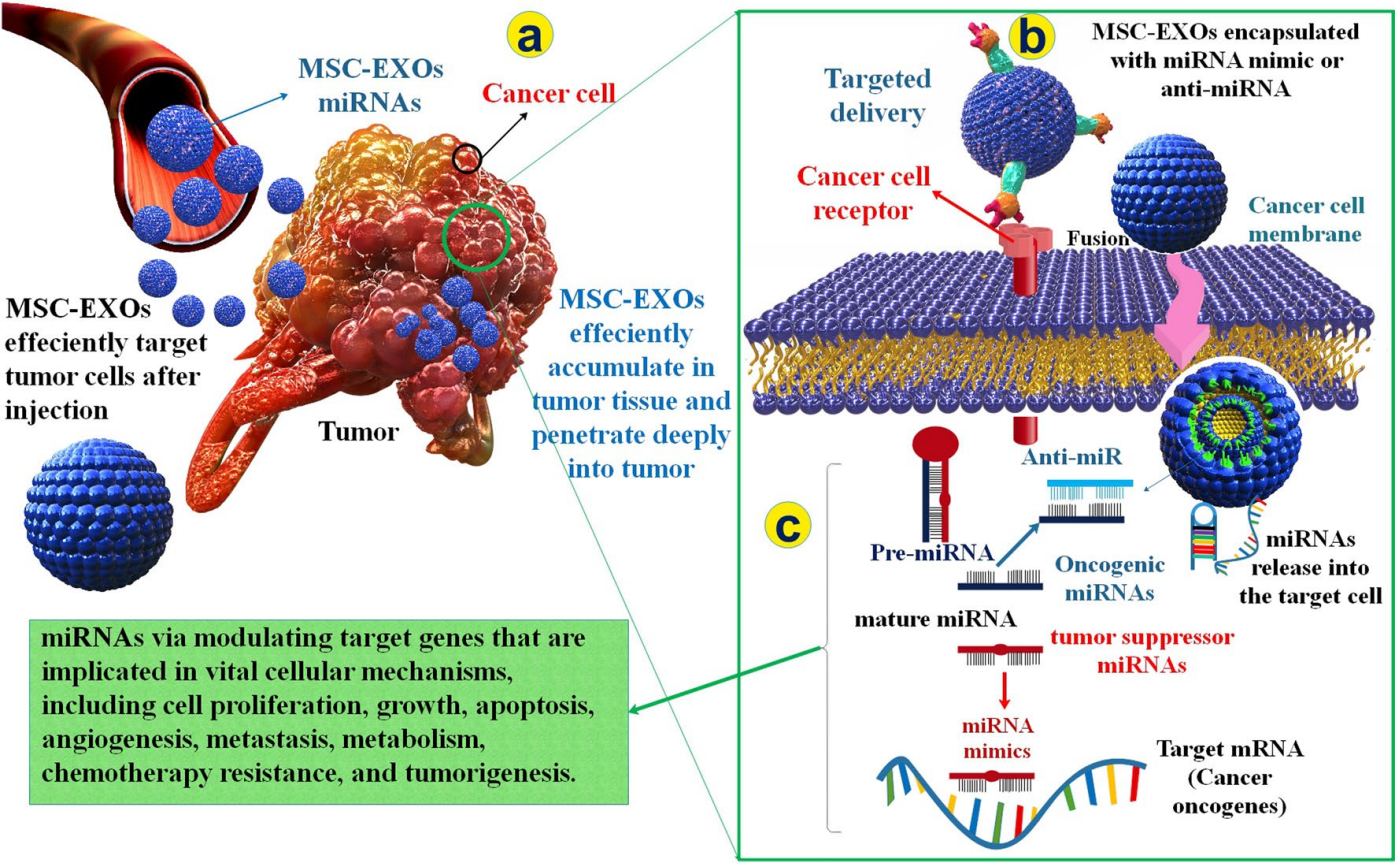
Function of MSC-EXOs as miRNA delivery method in different types of cancer**. a/b** Following discharge, MSC-EXOs are absorbed through cancer cells, **c** and the miRNAs encapsulated in MSC-EXOs regulate various processes, including involvement in cancer and tumor microenvironment immune responses, possibly tumor development, invasion, metastasis, angiogenesis, and chemotherapy resistance

Numerous investigations have displayed that MSC-EXOs play a significant function in tumor development, angiogenesis, malignancy, and medication resistance. However, inconsistent consequences have shown that MSC-EXOs could as well as inhibit tumors by particular mechanisms, including controlling immune reactions and intercellular signaling [118]. Unchanged MSC-EXOs can suppress tumors, while altered MSC-EXOs have participated in the inhibition of cancer development and expansion by the transfer of numerous therapeutics agents [119, 120]. In most cancers, the disorder in the regulation of miRNAs not only happens as a result of malignancy development, but is per se implicated in tumor suppression and progress because of their functions as oncomiRs or tumor suppressor miRNAs. MiRNA repair is commonly attained via increasing the expression level of tumor suppressors miRNAs utilizing synthetic miRNA mimics and viral carriers or even decreased expression level of oncomiRs utilizing antagomiRs [119] (Fig. 8).

BM-MSC-EXOs miR-205–5p exerts inhibitory efficacy on developing hepatocellular carcinoma (HCC) via controlling CDKL3. This miRNA expression was decreased while CDKL3 was increased in HCC. BM-MSCs-EXOs inhibited the cellular growth of HCC in vitro and in vivo. Loss of CDKL3 impaired the malignant processes of HCC cells and could even disturb the pro-tumor efficacy of downregulated BM MSC-EXOs miR-205–5p [121]. Recently, researchers used AD-MSC-EXOs to carry miR-381 mimic to MDA-MB-231 cells to clarify their effectiveness on triple-negative breast cancer cells. This miRNA encapsulated within AD-MSC-EXOs considerably decreases the expression of epithelial-to-mesenchymal transition (EMT) associated genes and proteins. Remarkably, this method suppressed the proliferation, migration, and malignancy capability of MDA-MB-231 and improved their apoptosis in vitro. As a result, this type of EXOs could be applied as an effective drug delivery system for miRNAs [122]. In another study, Jiang et al. showed that Exo-miR-7-5p isolated from BM-MSCs stimulated the organization of acute myeloid leukemia (AML) cells susceptible to apoptosis and a low viability amount, with OSBPL11 expression suppressed via the PI3K/AKT/mTOR signaling pathway. This method showed tumor-homing efficacy in vitro and in vivo and stopped AML progression [123]. Kurniawati et al. used BM-MSCs-EXOs to deliver miR-let-7c for suppression of the development of Castrate-resistant prostate cancer (CRPC). This investigation showed the tumor-preventative role of miR-let-7c in inhibiting cell proliferation and migration of CRPC-like cells [124]. In another study, researchers transfected BM-MSCs with a miR-146b expression plasmid, and isolated EXOs obtained via the MSCs. Intra-tumor administration of EXOs received from miR-146-expressing MSCs remarkably decreased glioma xenograft development in an animal model of early brain malignant growth [125] (Table 1).

**Table 1:** MSC-EXOs as miRNAs delivery system in cancer

MSC-EXOs	Type of cancer	miRNAs	miRNAs function	Route of administration	Explain	References
AD-MSC-EXOs	Bladder cancer	miR-138-5p	Tumor suppressor	Subcutaneous injections	This method is an efficient delivery carrier for small molecule medications in vivo, and EXOs-carried miR-138-5p is a hopeful curative factor for BC therapy	[166]
BM-MSC-EXOs	Liver cancer	miR-205–5p	Tumor suppressor	Subcutaneously injected	This technique has inhibitory efficacy on the development of liver cancer via controlling CDKL3	[121]
hUC-MSC-EXOs	Hepatoma	miR-451a	Tumor suppressor	–	HUC-MSC-EXOs miR-451a suppressed ADAM10 to inhibit the chemotherapy resistance, cell cycle transition, proliferation, cancer cell development, and progression, and increase apoptosis of Hepatoma cells	[167]
hUC-MSC-EXOs	Wilms tumor	miR-15a-5p	Tumor suppressor	Subcutaneously injected	This miRNA delivered via hUC-MSCs-Exo downregulates SEPT2 expression and inhibits WT cell development in vivo and in vitro	[168]
AD-MSC-EXOs	Triple-negative breast cancer (TNBC)	miR-381-3p	Tumor suppressor	–	EXOs-miR-381 suppressed the growth, migration, and invasion malignancy of breast cancer cells and increased their apoptosis in vitro	[122]
BM-MSC-EXOs	Acute myeloid leukemia (AML)	miR-7-5p	Tumor suppressor	Injected via a tail vein	Exo-miR-7-5p downregulates OSBPL11 via inhibiting the phosphorylation of the PI3K/AKT/mTOR signaling pathway, thus suppressing AML growth and increasing apoptosis	[123]
BM-MSC-EXOs	Breast cancer	LNA-anti-miR-142	OncomiRs	Intravenous injection	BM-MSC-EXOs can successfully transport anti-miR-142-3p to decrease the miR-142-3p and miR-150 rates and enhance the transcription of the regulative target genes (APC and P2X7R)	[169]
DP-MSC-EXOs	Breast carcinoma cells	miR-34a	Tumor suppressor	–	Genetically altered DP-MSCs were able to discharge of EXOs enriched with curative miRNAs and offered the possibility of use of EXO-based carrier for gene distribution	[170]
BM-MSC-EXOs	Castration-resistant prostate cancer (CRPC)	miR-let-7c	Tumor suppressor	–	These miRNAs can be effectively loaded into BM-MSC-EXOs. Therapy without carrier or MSC-EXOs-entrapped miR-let-7c led to remarkable decreases in cell proliferation and development in CRPC cells	[124]
BM-MSC-EXOs	Glioma	miR-146b	Tumor suppressor	Intratumoral injection	Administration of an intratumoral dose of 50 μg miR-146 loaded in BM-MSC-EXOs remarkably decreased glioma xenograft development in rat brains	[125]
BM-MSC-EXOs	Glioblastoma Multiforme	miR-9	OncomiRs	–	To inhibit miR-9, techniques were created with Cy5-tagged anti-miR-9. This method leads to enhanced apoptosis and caspase action	[171]

### MSC-EXOs as miRNAs delivery system in infectious diseases

The application of MSC-EXOs in the therapy of infectious diseases is a promising host-directed therapy. MSC-EXOs have an excellent capability for the treatment of inflammatory and infectious diseases. Moreover, MSC-EXOs have analogous capabilities to their parent cells, which have an extraordinary ability to regulate immune reactions because of their curative biomolecules [126, 127]. Furthermore, protein profiling of MSC-EVs shows that exosomal proteins are associated with the biological procedures, including the innate immune system, anti-bacterial, host-virus interplay, cellular oxidant detoxification, and complementarity and clotting cascades [128]. Infectious diseases, such as viral infections are related to changed amounts of host miRNAs. The efficacy of host miRNAs on viral infection can be used per se and indirectly. The direct effectiveness of miRNAs on virus control happens via directly binding various zones of the RNA virus genome. The indirect effectiveness comprises regulating a cellular transcript encoding a host agent required for one or more stages in viral replication. However, the virus may inhibit the production of miRNAs in an antiviral reaction [4, 129, 130]. In an investigation, researchers showed that UC-MSC-EXOs suppress Hepatitis C virus (HCV) infection in vitro, particularly viral reproduction, with less cell toxicity. The results showed that miRNAs from UC-MSC-EXOs had their exclusive expression profiles, and these functional miRNAs, mainly demonstrated via let-7f, miR-145, miR-199a, and miR-221 discharged from UC-MSC-EXO, widely involved in the inhibition of HCV RNA reproduction. Furthermore, UC-MSC-EXO treatment presented synergistic results when incorporated with U.S. FDA accepted interferon-α or telaprevir (VX-950), improving their anti-HCV capability and therefore enhancing the clinical importance of these regenerative ingredients for future usage as optimal adjuvants of anti-HCV treatment [131].

Moreover, UC-MSC-EXOs were isolated and applied to the in vitro acute lung injury (ALI) model. UC-MSC-EXOs restored the impaired alveolar fluid clearance of alveolar epithelial cells induced by influenza A (H5N1) infection. Still, this change was less statistically significant than the restoration by UC-MSCs. However, EXOs prevented changes in the alveolar protein permeability similarly to that of UC-MSCs [132].

In viral pneumonia, apart from the suppression of hypercytokinemia, inhibition of viral reproduction and attack on viruses are based pathways of MSC-EXOs treatment. MSC-EXOs miRNAs might bind to the viral genome to inhibit viral RNA transcription or protein translation vital for viral reproduction [133]. Sepsis is an acute deadly factor in COVID-19, and treatment via MSC-EXOs has improved the quantity of recovery in the sepsis animal model. At the same time, MSC-EXOs also suppressed the release of pro-inflammatory factors, such as TNF-α, IFN-γ, IL-6, IL-17, and IL-1β. They augmented the discharge of anti-inflammatory factors, containing IL-4, IL-10, and TGF-β. Furthermore, MSC-EXOs reduced the number of chemokines in the serum when injected. In another investigation, researchers utilized one dose, IV injection (15 mL) of allogeneic BM-MSC-EXOs (ExoFlo) to cure severe COVID-19. Consequently, 71 percent of the patients recovered, 13% remained extremely badly ill although stable, and 16% died for causes not related to this treatment method. Furthermore, laboratory quantities showed important improvement in absolute neutrophil amount and lymphopenia, with average CD3 + , CD4 + , and CD8 + cell amounts increasing [91]. In another investigation, Li and colleagues discovered the controlling pathway of miR-133 released from BM-MSC-EXO on myocardial fibrosis and EMT in viral myocarditis (VMC) animal model by regulating mastermind-like 1 (MAML1). EXOs were isolated and attained by ultracentrifugation, which was recognized via transmission electron microscope and western blot analysis. BM-MSC-EXO enhanced miR-133a expression in VMC rats and successfully enhanced the VMC rat cardiac action and myocardial fibrosis, enhanced cardiomyocyte viability, and suppressed the EMT procedure. Enhanced miR-133a in EXOs reinforced the developments. Inhibited miR-133a efficiently reversed the efficacy of BM-MSC-EXOs on VMC rats [134].

### MSC-EXOs as miRNAs delivery system in autoimmune and neurodegenerative diseases

In a study, investigators studied whether MSC-EXOs can carry miR-223-3p to remedy autoimmune hepatitis in an animal model. Researchers showed that MSC-EXOs was effectively combined with miR-223-3p and transported miR-223-3p into macrophages. Moreover, therapies of either naked EXOs or EXOs-miR-223-3p effectively decreased inflammatory reactions in the autoimmune chronic active hepatitis and IL-1, IL-6, and TNF-α discharge in both the liver and macrophages. The pathway may be associated with controlling miR-223-3p rate and STAT3 expression level in the liver and macrophages [135]. In another investigation, researchers showed that therapy of diabetic peripheral neuropathy in diabetic mice with MSC-EXOs-miR-146a for two weeks considerably augmented and reduced the nerve conduction speed and thermic and mechanical stimuli threshold, in order, while it took four weeks of EXO-naive therapy to attain this recovery. Contrasted with EXO-naïve, MSC-EXOs-146a considerably inhibited the peripheral blood inflammatory monocytes and the triggering of endothelial cells by suppressing the TLR-4/NF-κB signaling pathway [136]. BM-MSC-EXOs loaded with atorvastatin could show superior pro-angiogenic capability in diabetic wound healing. Besides, BM-MSC-EXOs-atorvastatin improved the proliferation, migration, tube organization, and VEGF rate of endothelial cells in vitro. MiR-221-3p was upregulated via atorvastatin-EXO induction, and miR-221-3p suppressor inhibited the pro-angiogenesis efficacy of atorvastatin-EXOs [137]. Furthermore, hBM-MSC-EXOs overexpressing miR-26a-5p postponed the harm of synovial fibroblasts in vitro and reduced osteoarthritis harm in vivo. Overall, this method used as an inhibitor for the damage of synovial fibroblasts by PTGS2 in osteoarthritis, which is of importance for the therapy of osteoarthritis in rats [138].

In another study, in vitro studies demonstrated that EXOs miR-146a released from BM-MSCs was delivered into astrocytic glial cells, and an enhanced rate of miR-146a and a reduced rate of NF-κB were detected in astrocytic glial cells. This investigation shows that exosomal delivery of miR-146a is implicated in the repair of cognitive disorder in an animal model of Alzheimer’s disease [139]. Qiang Li et al. used AD-MSCs-EXOs to deliver miR-188-3p for therapy-inhibited autophagy and pyroptosis while enhancing proliferation by binding to CDK5 and NLRP3 in mice and MN9D cells. It was shown that miR-188-3p could be a novel curative purpose for treating Parkinson's disease [140].

Damage to Retinal Ganglion Cells (RGC) and their axons is the primary reason for blindness. Researchers showed that MSC-EXOs were efficient in preserving RGC. And also, this method improved RGC survival and axon efficiency in the animal ocular nerve damage model while partially inhibiting RGC axon damage and dysfunction. To additionally study, the pathway of RGC preservation via MSC-EXOs, transfected MSCs with siRNA to inhibit the Argonaute-2 gene (the main miRNA effector) and separated the EXOs produced. It was found that the EXOs effectively carried their “payload” to the internal retina and that the efficacy was miRNA-related, with the curative efficacy of MSC-EXOs being decreased when Argonaute-2 was knocked out [141] (Table 2).

**Table 2:** MSC-EXOs as miRNAs delivery system in Autoimmune and Neurodegenerative Diseases

MSC-EXOs	Type of diseases	miRNAs	Route of administration	Explain	References
BM-MSC-EXOs	Diabetic peripheral neuropathy (DPN)	miR-146a (EXO-146a)	Intravenously injected via a tail vein	BM-MSCs-EXOs as biologic carriers of miR-146a can efficiently intercede and improve the curative action of MSCs in diabetic mice	[136]
HAD-MSC-EXOs	Diabetic wound	miR-21-5p	Injected intraperitoneally (i.p.)	This method improves the production and migration of keratinocytes by the Wnt/β-catenin pathway in vitro and speeds up diabetic wound recovery via enhancing re-epithelialization, collagen repair, angiogenesis, and vessel maturation in vivo	[172]
BM-MSC-EXOs	Autoimmune hepatitis	miR-223-3p	Injected intraperitoneally (i.p.)	BM-MSC-EXOs was effectively loaded with miR-223-3p and transported miR-223-3p into macrophages. Furthermore, there was the absence of side effects of EXOs on the macrophages	[135]
BM-MSC-EXOs	Alzheimer’s disease	miR-146a	Intracerebroventricular injection	Researchers showed that BM-MSCs ameliorate cognitive disorder in an Alzheimer’s disease model by enhancing the expression of microRNA-146a in a part of the limbic lobe	[139]
HUC-MSC-EXOs	Alzheimer’s disease	miR-223	–	HUC-MSC-EXOs miR-223 preserved neuronal cells from apoptosis via the PTEN-PI3K/Akt pathway and offered a powerful curative method for Alzheimer’s disease	[173]
AD-MSC-EXOs	Parkinson's disease	miR-188-3p	Injected intraperitoneally (i.p.)	AD-MSCs-EXOs to deliver miR-188-3p for therapy inhibited autophagy and pyroptosis while enhancing proliferation by binding to CDK5 and NLRP3 in mice and MN9D cells	[140]
synovial MSC-EXOs	Degenerative arthritis	miR-140-5p	Intra-articular injection	This method increased the proliferation and migration of articular chondrocytes without harming extracellular matrix release in vitro. In contrast, in vivo, SMSC-EXOs miR-140-5p effectively inhibited osteoarthritis in an animal model	[174]

### MSC-EXOs as miRNAs delivery system in other diseases

MSC-EXOs control vascular smooth muscle cell (VSMC) roles to suppress neointimal hyperplasia (NIH). Injection of hUC-MSC-EXOs inhibited NIH afterward artery ligation. HUC-MSC-EXOs reduced the intima and media region and intima/media proportion, enhanced the contractile phenotype protein SM22a in the media coating, and decreased Serpine1 expression in the carotid artery. MiR-148a-3p was improved in hUC-MSC-EXOs and inhibited Serpine1 via direct binding to its 3′-UTR area. Furthermore, hUC-MSC-EXOs miR-148a-3p inhibited VSMC phenotypic switching and migration via targeting Serpine1 [142]. The separated UC-MSC-EXOs had a characteristic cup-formed morphology, expressed the particular exosomal markers Alix, CD63, and TSG101, and were about 50–150 nm in size. TGFβ1 at 10 ng/ml considerably improved endometrial fibrosis, which was reversed via 20 µg/ml UC-MSC-EXOs. Exosomal miR-145-5p enhanced TGFβ1-stimulated endometrial fibrosis. UC-MSC-EXOs might reverse endometrial stromal cell fibrosis by controlling miR-145-5p/ZEB2 axis, demonstrating a possible new approach to augment endometrial regeneration [143]. In another investigation, MSCs were delivered with anti-let-7i-5p afterward, EXOs were separated and purified to carry anti-let-7i-5p oligonucleotides to suppress the rate of let-7i-5p in kidney tubular epithelial cells (NRK-52E). Furthermore, mice injected with MSC-EXOs anti-let-7i-5p exhibited decreased renal fibrosis and enhanced kidney function when challenged with a unilateral ureteral obstacle (UUO) [144]. In another study, 24 h afterward surgical treatment, BM-MSCs-EXOs-miR-146a-5p injection enhanced neurological function decreased apoptotic and neurodegenerative diseases and suppressed inflammatory reaction [145] (Table 3).

**Table 3:** MSCs-EXOs as miRNAs delivery system in different diseases

MSC-EXOs	Diseases	miRNAs	Route of administration	Explain	References
HUC-MSC- EXOs	Neointimal hyperplasia (NIH)	MiR-148a-3p	Intravenously injected	HUC-MSC-EXOs suppressed NIH in a mouse carotid artery ligation model, and the suppressor properties on VSMC phenotypic switching and migration interceded via transfer of miR-148a-3p to VSMCs to target Serpine1	[142]
UC-MSC- EXOs	Endometrial fibrosis	miR-145-5p/ZEB2	–	This method might reverse endometrial stromal cell fibrosis by controlling miR-145-5p/ZEB2 axis, showing a possible new approach to improve endometrial regeneration	[143]
MSC-EXOs	Renal fibrosis	anti-let-7i-5p	Intravenously injected	Anti-let-7i-5p from MSC-EXOs uses anti-fibrotic efficacy in TGF-β1-stimulated fibrogenic reactions in NRK52E cells in vitro also in the UUO-stimulated renal fibrosis model in vivo by triggering the TSC1/mTOR pathway	[144]
BM-MSC-EXOs	Myocarditis	miR-133	intraperitoneally injected	Increased exosomal miR-133a promoted cardiac action and prevent myocardial fibrosis, and EMT in rats with VMC also improves survival rate and suppresses apoptosis of cardiomyocytes in VMC by targeting MAML1	[175]
BM-MSC-EXOs	Intracerebral hemorrhage (ICH)	miR-146a-5p	Intrastriatal injection	This method could suggest neuroprotection and functional recovery afterward ICH by decreasing neuronal apoptosis and inflammation related to the suppression of microglial M1 polarization via a decrease expression of IRAK1 and NFAT5	[145]

## MSC-EXOs as miRNAs delivery system in the clinical stage

Up to now, an investigation on www.clinicaltrials.gov, which found 26 outcomes related to the keywords "Mesenchymal Stem Cells Exosomes". Moreover, clinical evaluations of MSC-EXOs are presently underway for insulin-dependent diabetes, cerebral infarction, COVID-19, Alzheimer's disease, and degenerative arthritis. However, of those studies, only one comprises what can be regarded as MSCs-EXOs as a medication transfer method: iExosomes in treating participants with metastatic pancreas cancer with KrasG12D mutation [146–148]. MSC-EXOs, as one of the most possible in vivo drug delivery systems, require to be additional studied and advanced via investigators [149]. For example, available pre-clinical information shows the efficient transfer of EXOs encapsulated with siRNA targeting KRASG12D resulting in tumor control in several mouse models of Pancreatic ductal adenocarcinoma (PDAC). Extensive generation of KRASG12D-siRNA loaded EXOs from MSCs will be implemented at the MD Anderson Cancer Center utilizing predefined GMP-compliant protocols. This is a single arm, single institute, phase I test assessing therapy with KRASG12D-siRNA loaded EXOs. This phase I trial investigates the most significant amount and adverse events of MSC-EXOs with KrasG12D siRNA (iExosomes) in curing patients with pancreatic malignant with KrasG12D mutation that has metastasized. iExosomes may work improved at curing patients [150]. Furthermore, a recently recorded trial (NCT04276987) aims to study the side effects and effectiveness of aerosol inhalation of allogenic AD-MSC-EXOs in patients with SARS-CoV-2 infection. The results demonstrate that a successive five days inhalation injection of clinical level hAD-MSC-EXOs up to a total quantity of 2.0 × 10^9^ nanovesicles were possible and well tolerated in seven SARS-CoV-2 patients, with no proof of pre-identified side effects, instant clinical inconsistency, or dosage-related toxicity at each of the amounts evaluated. This harmlessness profile is followed by CT scan recovery within seven days [151].

Currently, several investigations on novel or improved techniques for injecting curative miRNAs to the body, preserved transfer in the blood, targeted delivery of miRNAs to host cells, effective absorption via host cells, and improved gene targeting in the cell. MiRNAs as therapeutic agents have seldom been implicated in these clinical phases; however, numerous investigations have been done on miRNAs in MSC-EXOs in animal models. The clinical usage of miRNAs in the therapy of EXOs isolated from MSCs is a very significant investigation direction in the future [152, 153]. For example, in a study (NCT03562715), peripheral blood EXOs miRNA136, miRNA494, and miRNA495 genes expression in contrast to UC-MSC-EXOs in patients with pregnancy-associated hypertension and toxemia were detected. According to the recognized and recorded information, MSC-EXOs are going to be excellent biological vehicles for the treatment of different diseases. Moreover, it is encouraged to explore deeper into the possibility of MSC-EXOs between COVID-19 treatment and provide efficient therapy with maximum safety [154] (Table 4).

**Table 4:** Different MSC-EXOs in a clinical trial

MSCs-EXOs	Diseases	Clinical study stage	Participants	Injection route	Ref	Trial aim
MSC-EXOs (SY)	Pancreatic Cancer	Phase 1	28	Intravenously	NCT03608631 [150]	Mesenchymal stromal cells-derived exosomes with KRAS G12D siRNA
AD-MSCs-EXOs	COVID-19	Phase 1	24	Aerosol inhalation	NCT04276987 [151]	A pilot clinical study on inhalation of mesenchymal stem cells exosomes treating severe novel coronavirus pneumonia
MSC-EXOs	COVID-19	Phase 1 Phase 2	30	Inhalation	NCT04276987 [176]	Evaluation of safety and efficiency of method of exosome inhalation in SARS-CoV-2 associated pneumonia
Allogenic AD-MSCs-EXOs	Alzheimer disease	Phase 1 Phase 2	9	Inhalation (Nasal drip)	NCT04388982 [177]	the Safety and the Efficacy Evaluation of Allogenic Adipose MSC-Exos in Patients with Alzheimer's disease
UC-MSCs-EXOs	Macular holes (MHs)	Early Phase 1	44	Intravitreal injection	NCT03437759 [178]	Promoting healing of large and refractory macular holes (MHs)
Dermama-MSCs-EXOs	COVID-19	Phase 2 Phase 3	60	Intravenous route	NCT05216562	Efficacy and safety of EXOSOME-MSC (mesenchymal stem cell-derived exosomes) therapy to reduce hyper-inflammation in moderate COVID-19
WJ-MSCs-EXOs	Retinitis Pigmentosa	Phase 2 Phase 3	135	Subtenon injection	NCT05413148 [179]	The effect of stem cells and stem cell exosomes on visual functions in patients with retinitis pigmentosa
UC-MSCs-EXOs	Diabetes Mellitus Type 1	Phase 2 Phase 3	20	Intravenous	NCT02138331 [180]	Effect of microvesicles and exosomes therapy on β-cell Mass in Type I diabetes mellitus (T1DM)

## MSC-EXOs as a drug delivery system advantages and disadvantages

The use of MSC-EXOs is safe regarding adverse effects of MSC therapy, which may include consequences such as potential tumorigenesis by cell transplantation and obstruction in the distal vasculature by intravascular injection. The advantages are that MSC-EXOs can be mass-produced and sterilized via filtration and have a long shelf-life, but these properties do not extend to MSCs themselves [155].

**Fig. 9:**
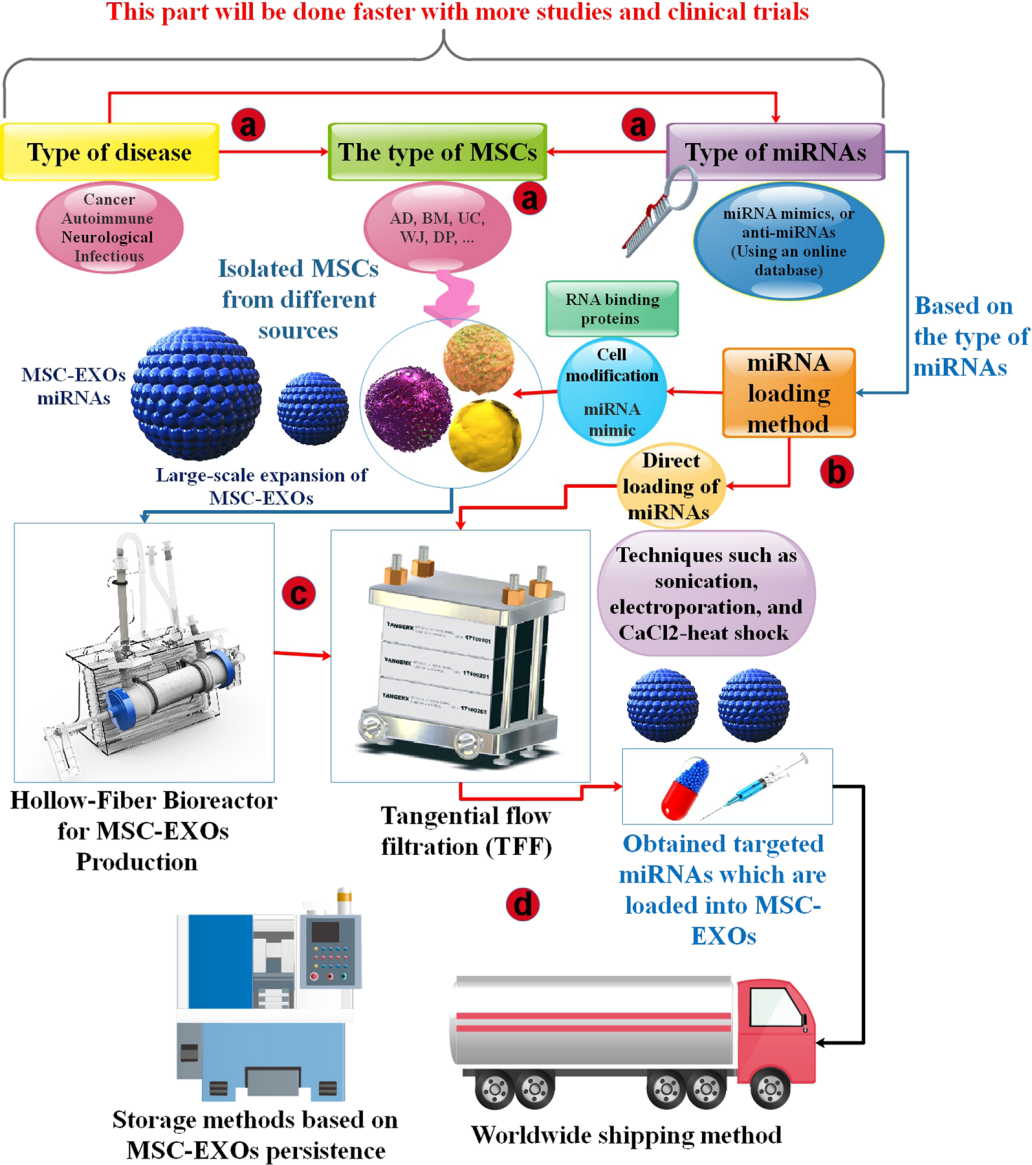
A road map for the large-scale production of various targeted miRNAs based on MSC-EXOs as a delivery system in different diseases. **a** Based on the type of diseases and microRNAs, we choose the appropriate and practical type of MSC (AD, BM, UC, WJ, and DP). **b** To load miRNAs in EXOs, two techniques have been used (cell modification for miRNA mimics and Direct loading of miRNAs). **c** After producing MSC-EXOs by hollow-Fiber Bioreactor, we need to purify them from other substances by tangential flow filtration (TFF) methods. **d** It is essential to establish proper storage methods and suitable transportation techniques for the mass production of MSC-EXOs containing miRNA

One restriction to utilizing EXOs to their completest possible is their confined release from cells, which constitutes the main blockage to effective, mass-scale EXOs generation. This is particularly true for MSCs, which, while capable to self-renew, have restricted development ability. MSCs tolerate aging afterward only a few passages, and EXOs isolated from aged MSCs have damaged regenerative capability compared to young MSCs [156–158]. Consequently, improving EXO generation is vital for both allogeneic and autologous EXOs-based treatments since 1- Time limitation, because EXOs-based therapies require to be injected as rapidly as possible, especially for cancer vaccination or to decrease the immunogenic efficacy arising from mismatched allogeneic cells; 2- high amounts of EXOs are needed for EXO-based treatments in a concise time, and while upscaling of cell cultures is a selection, this method is restricted via the growth kinetics and the number of cells separated from patients; and 3- developed EXOs synthesis would allow the generation of off-the-shelf curative [158, 159]. From interior to exterior, from physical situations to biomolecular parameters, investigators have offered techniques to enhance the generation of MSC-EXOs from different aspects. However, the methods suggested by the investigators will more or less influence the biological function of MSC-EXOs. And some of the effects could be uncontrollable and unverified. In the future, investigators may require to incorporate numerous techniques to form standardized and stable quality processes to offer strategies for large-scale generation of MSC-EXOs [149]. Furthermore, by comparing standard miRNA delivery systems, EXOs have the benefits, such as 1. EXOs are natural vehicles of cell generation and have excellent biocompatibility; 2. the outside of EXOs contains several types of proteins to inhibit the phagocytosis of macrophages and present the capability for a long-term circulation; 3. EXOs can avoid diverse biological obstacles and penetrate cells in different methods, including endocytosis; 4. EXOs can be packed with miRNA medications in an endogenous approach. Thus, EXOs can fill more miRNA as a therapeutic agent via exploring the endogenous loading method; 5. EXOs have homing potentials, which can be targeted via active and passive techniques [6] (Fig. 9).

Newly, it has been demonstrated that MSC-EXOs play an essential role in MSC-mediated paracrine effects through the delivery of miRNAs. Extracellular miRNAs exist with significant consistency in several body fluids and cell culture media in the vesicle-related form [160]. In addition, targeted miRNA delivery to tumors has been researched to target particular subcellular compartments, and receptor-interceded endocytosis is the most encouraging method. As hopeful cancer treatment methods, miRNAs are hard to pass through cell membranes due to their negative charge and lipophobic property. In addition, they are simply destruction next, entering the body. As high-quality carriers, MSC-EXOs can address this concern. In a related studies, the MSC-EXOs expressing miRNAs have been highlighted as crucial vehicles for gene or drug therapy [161]. The limitations of using this method include the identification of MSC-EXOs absence of uniform international standards, the low efficiency and expensive; traditional purification techniques need a long term to extract EXO, and available kits are costly. In addition, because the quality of EXOs is highly affected by temperature and time, the storage of EXOs is challenging [162]. In addition, refining the culture situation of MSCs will remarkably affect not only the generation efficiency but also the effectiveness of MSC-EXOs in consideration of the proteomic and genomic complications of EXOs. The culture situation alone may not be sufficient to address the further restrictions on the efficacy of MSC-EXOs. EXOs isolated from different cell kinds may have preferential targeting towards some cell kinds based on their membrane combination, therefore, imparting a differential effect on body systems [163].

## Conclusion

The use of EXOs as hopeful miRNAs delivery systems is remarkably associated with a trustworthy cell origin. Of the cell kinds recognized to release EXOs, the human MSC shows the most encouraging cell origin. MSC is not only a simply available cell kind that could be isolated from approximately all human tissues, and it is highly proliferative. One of the most attractive properties is the relative safety of MSCs. MSC-EXOs are therapeutically effective in animal models and display immunosuppressive action. MSC-EXOs have significant attributes that are examples of functional MSCs. Researchers showed the clinical effectiveness of MSC-EXOs for the treatment of different diseases. Currently, there are several studies are offering that MSC-EXO can be used for cancer therapy, gene therapy, medication delivery, regenerative medicine, and some other biomedical usages. Due to of some restrictions with the usage of MSCs themselves, such as controversial utilization in the presence of tumors, MSC-EXOs could be considered to be a cell-free alternative to intact MSCs. Moreover, the production of cell-based therapeutics is a challenging method. In contrast, MSC-EXOs can be simply stored at − 20 °C for six months without losing biological function. Therefore, EXO-based, cell-free therapies in regenerative medicine can be simpler to produce and prima facie safer. By EXOs, MSCs deliver their curative agents, particularly miRNAs, to target cells, and therein change gene expression and thereby improve therapeutic reaction. In addition, the results of the new miRNA study showed that some MSC-EXOs and MSCs had similar miRNA expression profiles, which is one of the causes why MSC-EXOs can replace MSCs for therapy. Present disease models are frequently mice or in vitro cell trials applied for cancer and other disease studies, which are relatively easy and absent information for comparative investigation with other disease models. Commonly, MSC-EXOs, as one of the most potential in vivo drug delivery systems, require to be additional studied and advanced via investigators.

## Data Availability

Not applicable.
